# Thermodynamic integration via differential evolution: A method for estimating marginal likelihoods

**DOI:** 10.3758/s13428-018-1172-y

**Published:** 2019-01-02

**Authors:** Nathan J. Evans, Jeffrey Annis

**Affiliations:** 1 Department of Psychology, University of Amsterdam, Amsterdam, Netherlands; 2 Department of Psychology, Vanderbilt University, Nashville, TN 37235 USA

**Keywords:** Marginal likelihood, Bayes factor, Bayesian model selection, Cognitive modeling

## Abstract

A typical goal in cognitive psychology is to select the model that provides the best explanation of the observed behavioral data. The Bayes factor provides a principled approach for making these selections, though the integral required to calculate the marginal likelihood for each model is intractable for most cognitive models. In these cases, Monte Carlo techniques must be used to approximate the marginal likelihood, such as *thermodynamic integration* (TI; Friel & Pettitt, *Journal of the Royal Statistical Society: Series B (Statistical Methodology), 70*(3), 589–607 2008; Lartillot & Philippe, *Systematic Biology, 55*(2), 195–207 2006), which relies on sampling from the posterior at different powers (called power posteriors). TI can become computationally expensive when using population Markov chain Monte Carlo (MCMC) approaches such as differential evolution MCMC (DE-MCMC; Turner et al., *Psychological Methods, 18*(3), 368 2013) that require several interacting chains per power posterior. Here, we propose a method called *thermodynamic integration via differential evolution* (TIDE), which aims to reduce the computational burden associated with TI by using a single chain per power posterior (*R* code available at https://osf.io/ntmgw/). We show that when applied to non-hierarchical models, TIDE produces an approximation of the marginal likelihood that closely matches TI. When extended to hierarchical models, we find that certain assumptions about the dependence between the individual- and group-level parameters samples (i.e., dependent/independent) have sizable effects on the TI approximated marginal likelihood. We propose two possible extensions of TIDE to hierarchical models, which closely match the marginal likelihoods obtained through TI with dependent/independent sampling in many, but not all, situations. Based on these findings, we believe that TIDE provides a promising method for estimating marginal likelihoods, though future research should focus on a detailed comparison between the methods of estimating marginal likelihoods for cognitive models.

When creating and testing psychological models, the goal is often to select the model among a pool of models that provides the best explanation of the observed data (Roberts & Pashler, 2000). Recent advancements in computing technology have led to an increasing number of formalized models (e.g., Ratcliff, 1978; Brown & Heathcote, 2008), which can produce precise quantitative predictions. The advantage of the precise, quantitative predictions of formalized models also comes with an additional challenge: How do we quantitatively choose the model that provides the best explanation of the psychological process? Although this choice may seem like an easy one, where the model that provides the best fit to the observed data should be selected, models that have a greater flexibility will often “over-fit” to the noise within the sample, leading to poor generalization (Myung & Pitt, 1997; Myung, 2000; Roberts & Pashler, 2000). Making this choice between models is a process known as model selection (Myung & Pitt, 1997). Traditional model selection methods such as the Akaike information criterion (AIC; Akaike, 1974) or the Bayesian information criterion (BIC; Schwarz, 1978) combine a goodness-of-fit statistic with a penalty term for model flexibility based on the number of parameters in the model. Although these methods are computationally simple, they can be inadequate in situations where, for example, parameters affect model flexibility differently (Myung & Pitt, 1997). In this article, we will use the Bayesian approach to model selection, an approach that balances goodness-of-fit and model flexibility in a cohesive framework (Myung & Pitt, 1997; Shiffrin et al., 2008).

The Bayesian approach to model selection is most easily introduced by first discussing the more familiar Bayesian approach to parameter estimation. The goal of Bayesian parameter estimation is to find the joint posterior distribution of the parameters, ***𝜃***, $p(\boldsymbol {\theta } |\boldsymbol {D},\mathcal {M})$, where $\mathcal {M}$ is the model, and ***D*** is the data vector. The joint posterior distribution is given by Bayes’ rule:

1 $$ p(\boldsymbol{\theta} |\boldsymbol{D},\mathcal{M}) = \frac{p(\boldsymbol{D} | \boldsymbol{\theta},\mathcal{M} ) p(\boldsymbol{\theta}|\mathcal{M})}{p(\boldsymbol{D}|\mathcal{M})}, $$

where $p(\boldsymbol {D} | \boldsymbol {\theta },\mathcal {M})$ is the likelihood function, $p(\boldsymbol {\theta }|\mathcal {M})$ is the prior probability of the parameters, and $p(\boldsymbol {D}|\mathcal {M})$ is the marginal likelihood found by marginalizing over all possible parameter values.

While Bayesian parameter estimation is primarily concerned with estimating the posterior distribution, $p(\boldsymbol {\theta }|\boldsymbol {D},\mathcal {M})$, the quantity of interest in Bayesian model selection is the marginal likelihood:

2 $$ p(\boldsymbol{D}|\mathcal{M}) = \int p(\boldsymbol{D} | \boldsymbol{\theta}) p(\boldsymbol{\theta}| \mathcal{M}) d\boldsymbol{\theta}. $$

The marginal likelihood can be used to perform Bayesian model selection by obtaining the posterior odds ratio:

3 $$ \frac{p(\mathcal{M}_{1} | \boldsymbol{D})}{p(\mathcal{M}_{2} | \boldsymbol{D})} = \frac{p(\boldsymbol{D} | \mathcal{M}_{1})}{p(\boldsymbol{D} | \mathcal{M}_{2})} \times \frac{p(\mathcal{M}_{1})}{p(\mathcal{M}_{2})}, $$

where the term $\frac {p(\boldsymbol {D} | \mathcal {M}_{1})}{p(\boldsymbol {D} | \mathcal {M}_{2})}$ is the Bayes factor and $\frac {p(\mathcal {M}_{1})}{p(\mathcal {M}_{2})}$ is the prior model odds. Bayesian model selection is often performed in the absence of the prior model odds. In this case, only the Bayes factor must be computed, a measure of evidence provided by the data in favor of one model over the other, which accounts for model flexibility by integrating over all possible parameter values (Myung & Pitt, 1997).

For very simple models with only a few parameters, sometimes the marginal likelihood integral can be analytically solved or estimated via standard numerical integration techniques. In many cases, where the model has more than a few parameters and the integral is intractable, we must resort to Monte Carlo techniques. The Monte Carlo approach relies on the relationship between certain forms of integrals and their corresponding representations as expected values, which can be approximated via sampling techniques (Evans & Brown, 2018).

One of the most straightforward Monte Carlo estimators of the marginal likelihood is the *arithmetic mean* estimator (Kass & Raftery, 1995; Evans & Brown, 2018), in which the average likelihood under samples from the prior is used as an approximation to the marginal likelihood. Although the arithmetic mean estimator is both conceptually simple and easy to implement, a large number of samples (e.g., 10,000,000+) is often required to obtain an accurate approximation of the marginal likelihood in a complex cognitive models (e.g., the linear ballistic accumulator; LBA; Brown & Heathcote, 2008) with around six parameters, and the number of samples required continues to increase with dimensionality (Evans & Brown, 2018). Thus, the arithmetic mean estimator is impractical for many complex cognitive models on standard computing hardware. Instead, graphical processing units (GPUs) capable of drawing a large number of samples in parallel can be used to obtain accurate estimates (Evans & Brown, 2018). Alternatively, there are a host of methods that can be used to approximate the Bayes factor or marginal likelihood using standard computing hardware such as bridge sampling (Gronau et al., 2017), the Savage–Dickey method (Wagenmakers et al., 2010), Chib’s method (Chib, 1995), the product method (Lodewyckx et al., 2011), the harmonic mean estimator (Gelfand & Dey, 1994), an adjusted arithmetic mean estimator (Pajor, 2017), or a generalization of the harmonic mean and inflated density ratio estimators (Wang et al., 2018). Note, the focus of this article will not be on comparing methods for marginal likelihood estimation. For those interested in a comparison of methods, we refer the reader to reviews by Friel and Wyse (2012) and Liu et al., (2016).

The present article will focus on improving an existing method known as *thermodynamic integration* (TI; Lartillot & Philippe, 2006; Friel & Pettitt, 2008). In TI, the posterior distribution is raised to powers between 0 and 1. Samples are drawn from each of these power posteriors and are then used to calculate the marginal likelihood. TI can be computationally expensive because sampling must be done over many power posteriors. This is especially true when using population Markov chain Monte Carlo (MCMC) techniques, such as differential evolution MCMC (DE-MCMC; ter Braak, 2006; Turner et al., 2013), to sample from the power posteriors. Specifically, DE-MCMC uses the differential evolution algorithm to generate proposals for the MCMC sampling process, where the newly proposed parameter values are informed by the difference in parameter values from two other random samples. This interaction between samples is obtained through simultaneously sampling from the posterior with a set of chains—the number of chains usually being 2–3 times the number of individual-level parameters—with the chains mutually informing the new proposals of one another. The DE-MCMC approach has become popular due to its ability to efficiently sample from models with correlated parameters (see Turner et al., 2013a, b, 2015; Evans & Brown, 2017; Evans et al., 2017b, 2018 for some applications), though it can be computationally burdensome when used in the context of TI as several chains are required for each power posterior, especially as the number of individual-level parameters grows.

Here, we present a variation of TI utilizing DE-MCMC in which only a single chain per power posterior is needed. Our method, which we call *TIDE*, implements TI within a population MCMC framework, an approach first introduced by Calderhead and Girolami (2009). We found that TIDE provided an approximation of the marginal likelihood that closely matched TI for models with a single subject. However, when extending the models hierarchically, we found that certain assumptions about the dependence between the individual- and group-level parameter samples resulted in large differences in the TI approximated marginal likelihood, where the standard dependent sampling results in higher marginal likelihoods than the recently implemented (e.g., Heathcote et al., 2018 implemented within their *DMC* package) independent sampling. We extended TIDE to these two different situations, with dependent sampling only requiring a natural extension of TIDE, and independent sampling adding the use of past iterations in a manner similar to “Z updating” from an extension of DE-MCMC, DE-MCz (ter Braak & Vrugt, 2008). We refer to the latter, independent sampling extension as *TIDEz*, and find that both TIDE and TIDEz can closely match to the marginal likelihood obtained through TI in some situations, but that this does not occur in all situations. However, when making inferences in our empirical data example, we find that both methods and sampling assumptions result in the same general inferences.

The remainder of this article will take the following format. First, we will discuss the TI method, and why TI can become computationally burdensome in some situations. Second, we will explain how integrating TI and DE-MCMC to form our new method, TIDE, can lead to a reduction in the computational burden associated with TI. Third, we present extensions of TIDE to hierarchical models, and show that they closely agree with the marginal likelihoods obtained by TI in some situations, using both simulated and empirical data.

## Thermodynamic integration

Thermodynamic integration (TI) (Friel & Pettitt, 2008; Lartillot & Philippe, 2006) is a method for estimating the marginal likelihood of a model. TI defines a set of posterior distributions. The likelihood of each posterior is raised to a power, *t*_*j*_ = {0,…,1} (called the *temperature*). These new posteriors are referred to as *power posteriors* and are defined as:

4 $$ p(\boldsymbol{\theta}|\boldsymbol{D},t_{j}) = \frac{p(\boldsymbol{D}|\boldsymbol{\theta})^{t_{j}}p(\boldsymbol{\theta})}{\int_{}^{} p(\boldsymbol{D} | \boldsymbol{\theta})^{t_{j}} p(\boldsymbol{\theta}) d\boldsymbol{\theta}}, $$

where *j* = {1,…,*k*} (called the *temperature rung*) indexes each of the *k* temperatures, dropping the model notation, $\mathcal {M}$, for brevity. The power posterior with a temperature of 0 is the prior distribution, and the power posterior with a temperature of 1 is the posterior distribution. After obtaining samples from each power posterior, ***𝜃***_*i*,*j*_ ∼ *p*(***𝜃***|***D***,*t*_*j*_), the average log-likelihoods, $\frac {1}{n} \sum _{i = 1}^{n}\ln p(\boldsymbol {D}|\boldsymbol {\theta }_{i,j})$, are computed. These form *k* points along a one-dimensional curve with respect to *t*, and the area under this curve is an estimate of the marginal likelihood. Since it is a one-dimensional curve, its area is easily estimated with standard numerical integration techniques. Friel and Pettitt (2008) suggest the trapezoidal rule:

5 $$\begin{array}{@{}rcl@{}} p(\boldsymbol{D}) &\approx& \sum\limits_{j = 2}^{k} \frac{t_{j}-t_{j-1}}{2}\\ &&\left[\frac{1}{n} \sum\limits_{i = 1}^{n}\ln p(\boldsymbol{D}|\boldsymbol{\theta}_{i,j}) \!+\! \frac{1}{n} \sum\limits_{i = 1}^{n}\ln p(\boldsymbol{D}|\boldsymbol{\theta}_{i,j-1}) \right]. \end{array} $$

TI relies on the discretization of temperatures, referred to as the *temperature schedule*. A temperature schedule in which *t*_*j*_ is set to the (*j* − 1)^*th*^ quantile of a *Beta*(*α*,1) distribution, where *α* = .3 has been shown to work well (Xie et al., 2010; Friel & Pettitt, 2008):

6 $$ t_{j} = (\frac{j-1}{k-1})^{\frac{1}{\alpha}}, $$

where *k* is the total number of temperatures, *j* = {1,…,*k*}. More about TI and its implementation can be found in Annis et al., (2018), and the exact mathematical details of TI can be found in the Appendix.

Notice that TI suffers from two major sources of error, the discretization of temperatures, and MCMC error. Discretization error can be reduced by increasing the number of temperature rungs, and MCMC error can be reduced by increasing the number MCMC samples per temperature rung. Although error reduction in TI is straightforward, increasing the number of MCMC samples and temperature rungs leads to increased computational workload.

Thus, a major drawback of the method is the computational burden that TI can impose in order to obtain accurate marginal likelihood estimates. For example, in prior work (Annis et al., 2018), we found the number of temperatures needed to obtain a stable estimate of the marginal likelihood to be around 20–35 for hierarchical LBA models. This computational burden increases when a population MCMC algorithm is used to obtain samples, where several interacting chains are necessary for each power posterior. The population MCMC approach we focus on is differential evolution MCMC (DE-MCMC; ter Braak, 2006; Turner, Sederberg, et al., 2013), which has become popular in cognitive psychology due to its ability to efficiently sample from posteriors with correlated parameters. It requires a number of chains equal to 2–3 times the number of parameters in the largest updating block, which within hierarchical models is commonly the number of parameters per individual. In the next section, we propose a method based on DE-MCMC that aims to reduce the number of chains needed for each power posterior to one.

## Thermodynamic integration via differential evolution (TIDE)

The algorithm we present combines TI and DE-MCMC so that only a single chain needs to be run for each power posterior, which provides the benefits of DE-MCMC while avoiding the costly overhead of having to run several chains per power posterior. The algorithm relies on the same approach as Calderhead and Girolami (2009) who originally proposed implementing TI within a population MCMC framework. Here, we propose an extension we refer to as *thermodynamic integration via differential evolution* (TIDE).

DE-MCMC uses the genetic algorithm, *differential evolution*, to generate proposals. Generally, genetic algorithms are a class of algorithms that involve some amount of *crossover* between different existing elements to create new elements, as well as some possible random *mutations* that change the new elements. DE-MCMC uses a set of interacting chains that are all simultaneously sampling from the posterior. To create a new proposal on iteration *i* for chain *c*, ***𝜃***_*i*,*c*_, the previous value on that chain, ***𝜃***_*i*− 1,*c*_, is added to the difference between two randomly chosen other chains, *l* and *m* (i.e., a crossover):

7 $$ \boldsymbol{\theta}_{i,c} = \boldsymbol{\theta}_{i-1,c} + \gamma(\boldsymbol{\theta}_{i-1,l} - \boldsymbol{\theta}_{i-1,m}) + \epsilon, $$

where *γ* controls the size of “jump” for the new proposal, and *𝜖* is a small amount of random noise (i.e., a mutation). The proposal is then accepted or rejected according to the Metropolis Hastings step, and the process continues on to create a proposal for the next chain. DE-MCMC forms a population of interacting chains, **Θ** = (***𝜃***_1_,..,***𝜃***_*C*_), where *C* is the total number of chains, with the following product distribution:

8 $$ p(\boldsymbol{\Theta}|\boldsymbol{D}) = \frac{1}{\prod_{c = 1}^{C} \int p(\boldsymbol{D} | \boldsymbol{\theta}_{c}) p(\boldsymbol{\theta}_{c}) d\boldsymbol{\theta}} \prod\limits_{c = 1}^{C} p(\boldsymbol{D}|\boldsymbol{\theta}_{c}) p(\boldsymbol{\theta}_{c}) $$

***𝜃***_*i*,*c*_ ∼ *p*(***𝜃***|***D***). This is the typical case in which samples are drawn from the posterior distribution. It is also possible to sample from a power posterior at temperature, *t*_*j*_. In this case, the population of chains forms the following product distribution:

9 $$ p(\boldsymbol{\Theta}|\boldsymbol{D},t_{j}) = \frac{1}{\prod_{c = 1}^{C} \int p(\boldsymbol{D} | \boldsymbol{\theta}_{c})^{t_{j}} p(\boldsymbol{\theta}_{c}) d\boldsymbol{\theta}} \prod\limits_{c = 1}^{C} p(\boldsymbol{D}|\boldsymbol{\theta}_{c})^{t_{j}} p(\boldsymbol{\theta}_{c}), $$

where ***𝜃***_*i*,*c*_ ∼ *p*(***𝜃***|***D***,*t*_*j*_). This is what we refer to as *standard TI with DE-MCMC* or just *standard TI*. A drawback of this approach is that it requires *C* chains for each power posterior. To more elegantly combine DE-MCMC with TI, we associate each chain, *c*, with a temperature, *j*, meaning that the index *c* can be dropped. TIDE then forms a population of interacting chains, **Θ** = (***𝜃***_1_,..,***𝜃***_*k*_), where each chain is associated with temperature, *j*:

10 $$ p(\boldsymbol{\Theta}|\boldsymbol{D},\boldsymbol{t}) = \frac{1}{\prod_{j = 1}^{k} \int p(\boldsymbol{D} | \boldsymbol{\theta}_{j})^{t_{j}} p(\boldsymbol{\theta}_{j}) d\boldsymbol{\theta}} \prod\limits_{j = 1}^{k} p(\boldsymbol{D}|\boldsymbol{\theta}_{j})^{t_{j}} p(\boldsymbol{\theta}_{j}). $$

where ***𝜃***_*i*,*j*_ ∼ *p*(***𝜃***|***D***,*t*_*j*_). Thus, TIDE only requires a single chain per power posterior by allowing chains to interact *between* power posteriors instead of *only within* power posteriors. After sampling, the average log-likelihood under each chain is computed, $\frac {1}{n} {\sum }_{i = 1}^{n}\ \ln p(\boldsymbol {D}|\boldsymbol {\theta }_{i,j})$, and the trapezoidal rule is used to estimate the marginal likelihood. It should also be noted that TIDE is equally applicable to any other method that approximates the marginal likelihood using power posteriors, such as TI “corrected”, or steppingstone sampling (SS; see Annis et al., 2018 for a tutorial on both of these methods). Next, we compare the performance of TIDE to standard TI for the data of an individual simulated subject, and extend it to hierarchical models for groups of subjects, and compare its performance to standard TI for a group of simulated subjects.

Algorithm 1 displays pseudo-code for how to implement TIDE. Once starting points for the parameter values (i.e., ***𝜃***_1_) are obtained, an iterative process is performed to obtain the posterior samples (**for***i* ← 2 **to***n***do**), which is performed for each temperature (**for***j* ← 1 **to***k***do**). Each iteration for each temperature requires selecting two other random chains (i.e., two other temperatures), creating the DE proposal using those selected chains, and then deciding whether to accept the proposal based upon the Metropolis Hastings step. After the iterative process is complete, the marginal likelihood estimate is obtained using the trapezoidal rule. 
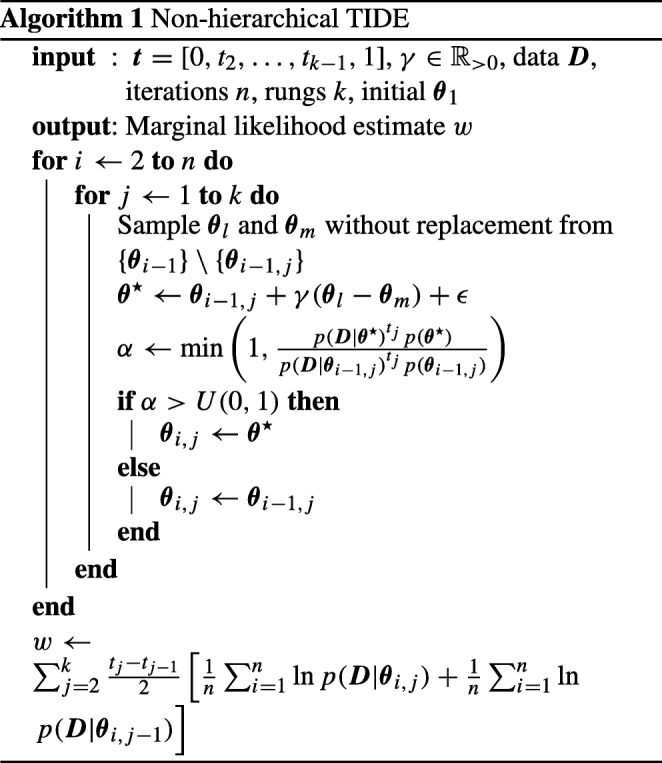


### TIDE for hierarchical models

Hierarchical models have become an increasingly popular method within cognitive psychology for making inferences on groups of participants (Shiffrin et al., 2008). Hierarchical models involve estimating parameters for individual participants (i.e., the *individual-level*, ***𝜃***), and constraining the estimates of each parameter for all individuals to follow a group-level distribution of the parameters (i.e., the *group-level*, ***ϕ***). Hierarchical models provide key benefits over non-hierarchical estimation, allowing information from different individuals to constrain the estimation of one another (commonly known as “shrinkage”), and providing a method of performing group-level inference on the entire dataset from experiments. Formally, a general hierarchical model can be defined as:

11 $$\begin{array}{@{}rcl@{}} \boldsymbol{D}_{s} &\sim& p(\boldsymbol{D}_{s} | \boldsymbol{\theta}_{s}) \\ \boldsymbol{\theta}_{s} &\sim& p(\boldsymbol{\theta}_{s} | \boldsymbol{\phi}) \\ \boldsymbol{\phi} &\sim& p(\boldsymbol{\phi}), \end{array} $$

where *s* indexes the participant. For hierarchical models of this form, the power posterior is given by:

12 $$ p(\boldsymbol{\theta},\boldsymbol{\phi}|\boldsymbol{D},t_{j}) = \frac{p(\boldsymbol{D}|\boldsymbol{\theta},\boldsymbol{\phi})^{t_{j}}p(\boldsymbol{\theta} , \boldsymbol{\phi})}{\int \int_ p(\boldsymbol{D}|\boldsymbol{\theta},\boldsymbol{\phi})^{t_{j}}p(\boldsymbol{\theta} , \boldsymbol{\phi}) d\boldsymbol{\theta} d\boldsymbol{\phi}}. $$

TIDE now forms two populations of interacting chains, **Θ** = (***𝜃***_1_,..,***𝜃***_*k*_) and **Φ** = (***ϕ***_1_,..,***ϕ***_*k*_), with the following product distribution:

13 $$\begin{array}{@{}rcl@{}} p(\boldsymbol{\Theta}, \boldsymbol{\Phi} |\boldsymbol{D},\boldsymbol{t}) &=& \frac{1}{\prod_{j = 1}^{k} \int \int p(\boldsymbol{D}|\boldsymbol{\theta}_{j})^{t_{j}} p(\boldsymbol{\theta}_{j} , \boldsymbol{\phi}_{j}) d\boldsymbol{\theta} d\boldsymbol{\phi}}\\ &&\prod\limits_{j = 1}^{k} p(\boldsymbol{D}|\boldsymbol{\theta}_{j})^{t_{j}} p(\boldsymbol{\theta}_{j} , \boldsymbol{\phi}_{j}) \end{array} $$

where (***𝜃***_*i*,*j*_,***ϕ***_*i*,*j*_) ∼ *p*(***𝜃***,***ϕ***|***D***,*t*_*j*_). After drawing samples from the joint distribution, the individual-level samples are used to compute the average likelihoods in the same way as before, $\frac {1}{n} \sum _{i = 1}^{n}\ \ln p(\boldsymbol {D}|\boldsymbol {\theta }_{i,j})$, which are in turn are used in the trapezoidal rule to obtain an estimate of the marginal likelihood. Although samples are drawn from the joint distribution, *p*(***𝜃***,***ϕ***|***D***,*t*_*j*_), only the individual-level samples, ***𝜃***_*i*,*j*_, are needed in the computation of marginal likelihood estimate. Thus, the group-level priors only enter indirectly into the estimation of the marginal likelihood by constraining the ***𝜃***_*i*,*j*_ samples. A proof is given in the Appendix.

Algorithm 2 displays pseudo-code for how to implement hierarchical TIDE. The algorithm is very similar to the non-hierarchical TIDE in Algorithm 1, though the iterative process now involves two stages: updating the group-level parameters, and updating the individual-level parameters. The first stage, updating the group-level parameters, is similar to updating the parameters in non-hierarchical TIDE, and requires selecting two other random chains (i.e., two other temperatures), creating the DE proposal using those selected chains, and then deciding whether to accept the proposal based upon the Metropolis Hastings step. Note that the Metropolis Hastings step shown here does not involve the probability of the data under the individual-level parameters (i.e., *p*(***D***|***𝜃***)), as the proposal does not involve new individual-level parameters, and therefore, the terms involving identical individual-level parameters cancel out in the Metropolis Hastings step. The second stage, updating the individual-level parameters, involves a loop over participants (**for***s* ← 1 **to***P***do**), as the parameters for each individual are updated separately to reduce dimensionality. After this, the process involves the same steps as stage one, though for the individual-level parameters for this participant. Also note that the Metropolis Hastings step shown here does not involve the prior probability of the group-level parameters (i.e., *p*(***ϕ***)), for the same canceling out reasons as above. 
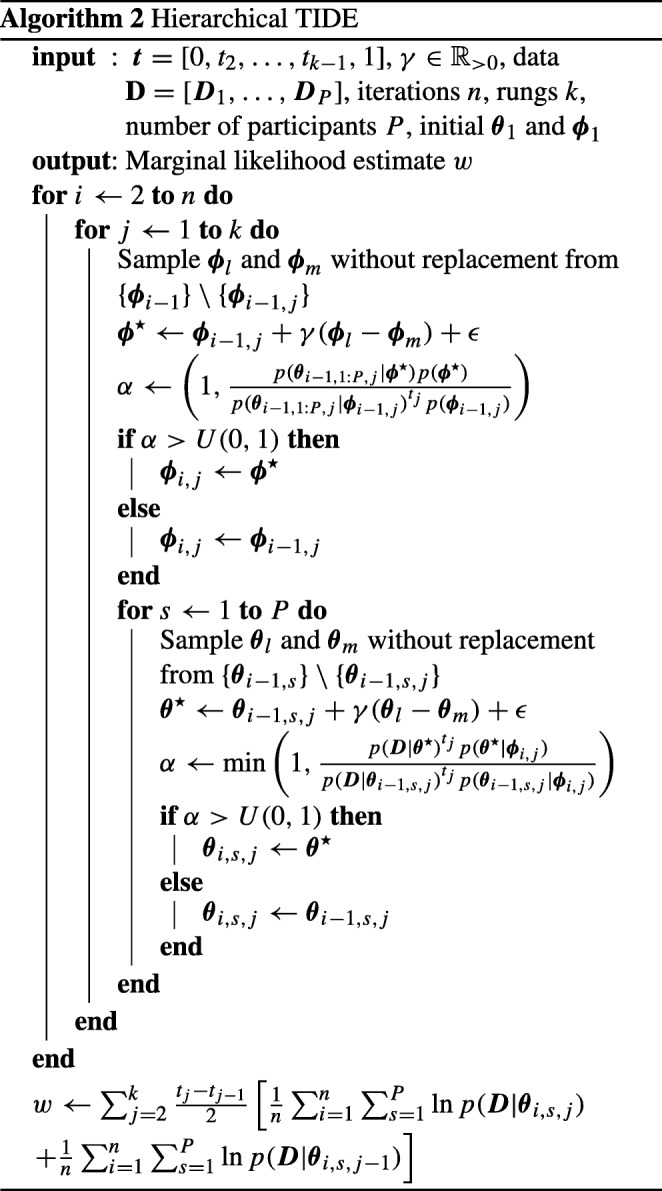


### Thermodynamic integration via differential evolution with Z updating (TIDEz)

Although hierarchical models are conceptually simple to implement, practical difficulties have been reported when extending cognitive models to these hierarchical structures. For example, when hierarchically estimating the diffusion model (Ratcliff, 1978), some researchers have recommended fixing specific parameters to only be estimated at the group level (i.e., all individuals share the same parameter value), due to these parameters having only small influences on the model likelihood, which results in difficulties accurately estimating the full hierarchical structure (e.g., Wiecki et al., 2013; the group-level fixing of inter-trial variability parameter is implemented in their *HDDM* package). A related problem has been referred to as the “zero-variance trap”, where all participants values for a certain parameter—a parameter that only has a small effect on the likelihood, and therefore, can be highly influenced by the group-level constraints—converge to a single value, resulting in the group-level variability between participants approaching zero, and the values becoming “stuck”. However, when using the DE-MCMC framework and a specific system of “blocking” outlined below, a simple solution can be used to remedy this problem by “breaking the dependency” between the individual and group-level parameters (Turner et al., 2013a, b; Tillman et al., 2017; Osth et al., 2018).

When using the DE-MCMC algorithm, the parameters being updated at any one point are usually split into different sampling blocks, as large numbers of parameters (i.e., high dimensionality) can lead to inefficient sampling. These blocks involve only specific parameters having proposals generated, and the posterior likelihood being conditioned on the other parameters. Commonly, a different block is used for the parameters of each participant, and for the group-level parameters. This means that the parameters of each individual participant are updated according to:

14 $$ p(\boldsymbol{\theta}_{i,s} | \boldsymbol{\phi}_{i}, \boldsymbol{D}) \propto p(\boldsymbol{D} | \boldsymbol{\theta}_{i}) p(\boldsymbol{\theta}_{i}|\boldsymbol{\phi}_{i}), $$

where *s* indexes the participant and *i* indexes the current sample. The parameters of the group are updated according to:

15 $$ p(\boldsymbol{\phi}_{i} |\boldsymbol{\theta}_{i},\boldsymbol{D}) \propto p(\boldsymbol{\theta}_{i}|\boldsymbol{\phi}_{i})p(\boldsymbol{\phi}_{i}). $$

Importantly, this system of blocking allows for a simple method of “breaking the dependence” between the individual- and group-level parameters, which has been found to remedy some of the practical issues that can occur in hierarchical models discussed above (Turner et al., 2013a, b; Tillman et al., 2017; Osth et al., 2018). Specifically, the method involves randomly pairing the values of the ***𝜃*** and ***ϕ*** parameters of different chains for the purposes of updating: for the updating of the ***𝜃*** parameters for a specific chain, the ***ϕ*** parameters from another random chain are selected to be conditioned on, and vise-versa^1^. Equation 14 can then be changed to:

16 $$ p(\boldsymbol{\theta}_{i,s} | \boldsymbol{\phi}_{l}, \boldsymbol{D}) \propto p(\boldsymbol{D} | \boldsymbol{\theta}_{i}) p(\boldsymbol{\theta}_{i}|\boldsymbol{\phi}_{l}), $$

where *i* indexes the current sample (i.e., the current chain), and *l* indexes another random chain. Likewise, Eq. 15 can then be changed to:

17 $$ p(\boldsymbol{\phi}_{i} |\boldsymbol{\theta}_{l},\boldsymbol{D}) \propto p(\boldsymbol{\theta}_{l}|\boldsymbol{\phi}_{i})p(\boldsymbol{\phi}_{i}). $$

Essentially, this random pairing “breaks the dependence” between the individual- and group-level parameters, resulting in the joint posterior having independent samples of individual- and group-level parameters, as opposed to the standard sampling method, which contains a full joint posterior. It should also be noted that the random pairing is performed as sampling without replacement: that this, each individual-level chain is randomly paired with one other group-level chain. However, this simple solution is no longer possible with TIDE, as each chain estimates the power posterior for a different temperature, and therefore, a different target distribution.

Although we cannot sample across *chains*, we can sample across *time*. This solution is similar to an updating procedure called Z-updating in DE-MCz (ter Braak & Vrugt, 2008) and so we refer to this algorithm as TIDEz. As discussed previously, the ***𝜃*** and ***ϕ*** parameters are updated separately, in different blocks. TIDEz randomly pairs the ***𝜃*** and ***ϕ*** samples using previous posterior samples. Equation 14 can then be changed to:

18 $$ p(\boldsymbol{\theta}_{i,s} | \boldsymbol{\phi}_{z}, \boldsymbol{D}) \propto p(\boldsymbol{D} | \boldsymbol{\theta}_{i}) p(\boldsymbol{\theta}_{i}|\boldsymbol{\phi}_{z}), $$

where *i* indexes the current sample, and *z* indexes a random previous posterior sample. Likewise, Eq. 15 can then be changed to:

19 $$ p(\boldsymbol{\phi}_{i} |\boldsymbol{\theta}_{z},\boldsymbol{D}) \propto p(\boldsymbol{\theta}_{z}|\boldsymbol{\phi}_{i})p(\boldsymbol{\phi}_{i}). $$

Note that we only use previous posterior samples after a certain number of initial iterations (i.e., not immediately, when the parameters may be a long way from the posterior), and we only reach a certain maximum number of iterations into the past. This introduces two extra “tuning” parameters that need to be set for the TIDEz algorithm: When the Z update starts (“*zStart*”), and the maximum number of iterations that can be reached into from the past (“*zLag*”). In our applications here, we set *zStart* to 2000, and *zLag* to 250.

Algorithm 3 displays pseudo-code for how to implement hierarchical TIDEz. The algorithm is almost identical to the hierarchical TIDE in Algorithm 2, with two key exceptions. Firstly, the iterative process shown here starts at *zStart*, as iterations before this work in an identical manner to hierarchical TIDE. Secondly, in the line before the creation of the DE proposal, the previous iteration of the individual-level/group-level parameter to pair with the current group-level/individual-level update is chosen, based on the *zLag* value. 
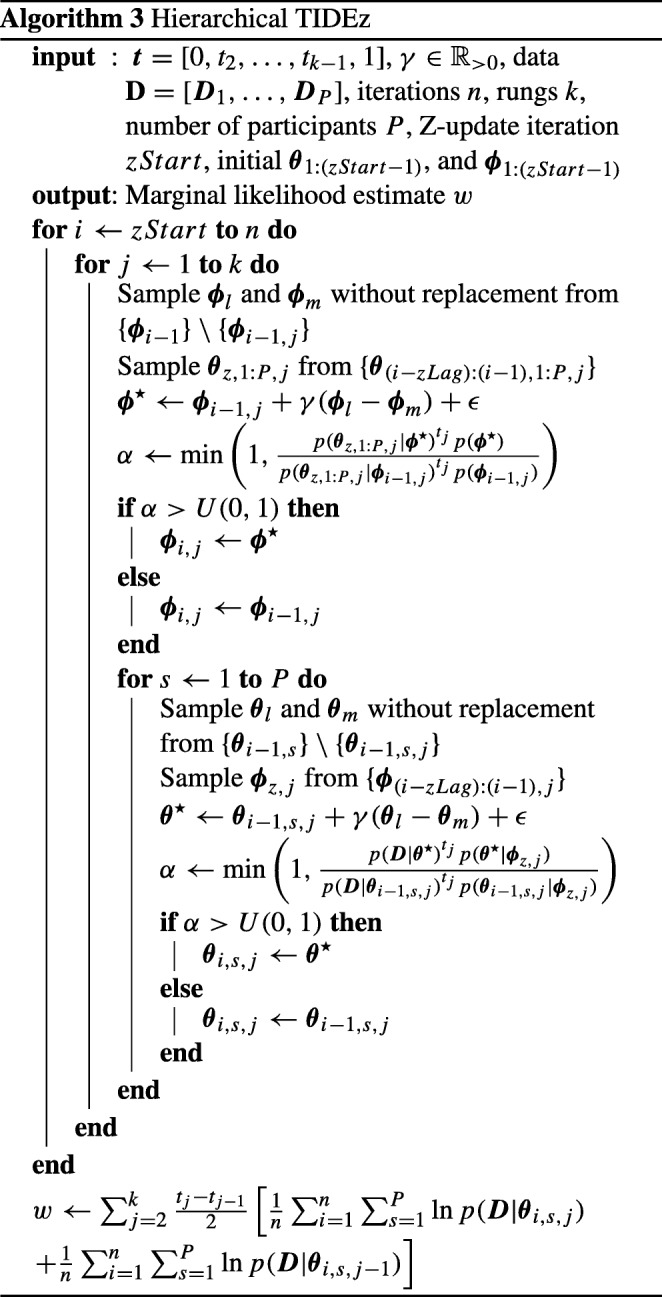


## Examples

### Individual subjects

Here, we use TIDE to approximate the marginal likelihood for a cognitive model. Specifically, we apply TIDE to the linear ballistic accumulator (LBA; Brown & Heathcote 2008), a commonly used model of decision-making (Forstmann et al.,^2008^, ^2011^; Brown et al., 2008; Donkin et al., 2009; Ho et al., 2014; Rae et al., 2014; Evans et al. 2017b, 2018). We use the LBA as a running example because it has an analytically tractable likelihood function, making computation of TIDE quick enough to allow for estimation to be performed within a short time-frame, and variances across independent estimates to be obtained. In addition, the LBA has been the applied model used in previous manuscripts on estimating marginal likelihoods (Evans & Brown, 2018; Annis et al., 2018), allowing a clear comparison to previous research. We begin by briefly explaining the LBA, before assessing TIDE in the case of data from an individual subject.

The LBA is a model of decision-making that falls within a class of models known as evidence accumulation models (Stone, 1960; Ratcliff, 1978; Ratcliff & Rouder, 1998; Brown & Heathcote, 2008). Evidence accumulation models propose that decision-making is the result of the accumulation of evidence for the different choice alternatives until the evidence for one alternative reaches a threshold and a decision is triggered. Specifically, the LBA proposes that this accumulation process involves independent racing accumulators for each alternative, with the rate of evidence accumulation being constant within a decision, but differing between decisions according to a normal distribution, truncated at 0. Evidence is also proposed to start at a random point for each accumulator that differs between decisions, with the starting evidence being uniformly distributed between 0 and some point less than the decision threshold. Lastly, the model contains some amount of time dedicated to processes outside of the decision, such as perception and motor responding. This results in the model having five parameters per accumulator: the mean rate of evidence accumulation over decisions (*v*), the standard deviation of evidence accumulation over decisions (*s*), the threshold amount of evidence required to make a decision (*b*), the upper bound of the uniform distribution of starting evidence (*A*), and the time dedicated to non-decision-related components (*t*0). However, in many applications of the LBA all parameters except for the mean and standard deviation in drift rate are constrained to have the same value for both accumulators, and the standard deviation for one accumulator is fixed to 1 to satisfy a scaling property within the model (Donkin et al., 2009), meaning that the model is commonly implement with six total parameters: the mean drift rate for the response alternative that matches the stimulus (*v*.*c*), the mean drift rate for the response alternative that does not match the stimulus (*v*.*e*), the standard deviation in drift rate for the response alternative that does not match the stimulus (*s*.*e*), *b*, *A*, and *t*0.

Specifically, we used the same simulated dataset as Evans and Brown (2018) and Annis et al., (2018). This dataset had two “within-subjects conditions” simulated, with a generating process that had no parameters differing between the conditions, which we call a “simple” dataset. As with Evans and Brown (2018) and Annis et al., (2018), we fit two models to each of these datasets: a “simple” model that constrained all parameters to take the same values over conditions, and a “complex” model that allowed mean drift rate, threshold, and non-decision time to vary over conditions. The specific model definition for data generation and fitting can be found in the Appendix.

**Fig. 1:**
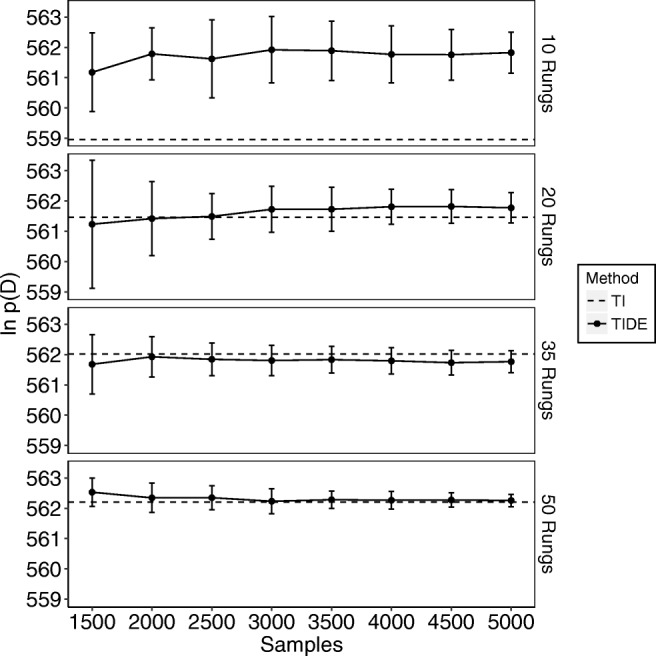
The estimated natural logarithm of the marginal likelihoods (*y*-axis) for the “simple” model across different numbers of samples (*x*-axis; note that this includes the samples discarded for burn-in) and different numbers of temperatures used (different plots: known as “rungs”). The *dashed lines* display the values obtained through standard TI, which used a fixed number of samples, and the *solid lines* display the values obtained through TIDE. *Error bars* are standard deviations based on ten replications

**Fig. 2:**
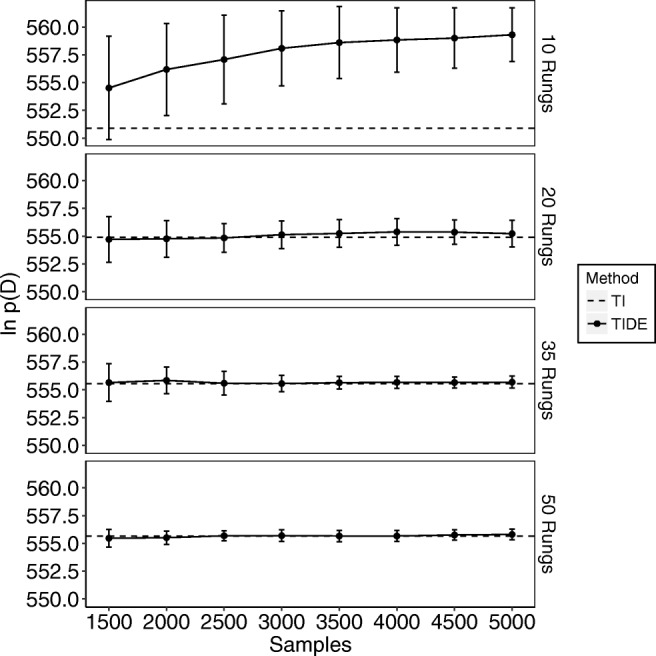
The estimated natural logarithm of the marginal likelihoods (*y*-axis) for the “complex” model across different numbers of samples (*x*-axis; note that this includes the samples discarded for burn-in) and different numbers of temperatures used (different plots: known as “rungs”). The *dashed lines* display the values obtained through standard TI, which used a fixed number of samples, and the *solid lines* display the values obtained through TIDE

The resulting log-marginal likelihood estimates for TIDE (solid lines) can be seen in Fig. 1 for the simple data set and Fig. 2 for the complex data set. For TIDE, we discarded the first 1,500 samples for each chain (i.e., each temperature) as burn-in, meaning that the *x*-axis of Figs. 1 and 2 begin at the end of burn-in. We compare these to the estimates obtained by standard TI (dashed lines), which used 12 chains for the simple model and chains 18 chains for the complex model (i.e., twice the number of free parameters), with 2300 samples per chain and the first 300 samples per chain discarded as burn-in. In terms of computational workload, TI used 27,600 samples per temperature for the simple model and 41,400 samples per temperature for the complex model, whereas TIDE (at the maximum point of the *x*-axis) used 5000 samples per temperature for each model,^2^ meaning that TIDE took 5.52 times (simple model) and 8.28 times (complex model) less samples than TI in this example (see the Appendix [Equation C3] for a more detailed theoretical comparison of computational workloads). Points are means based on ten independent replications and error bars are standard deviations. Each panel of the plot corresponds to the marginal likelihood estimate obtained with a given numbers of temperature rungs (10, 20, 35, and 50). The x-axis provides the number of samples taken from the power-posterior of each temperature, and the *y*-axis is the estimated log-marginal likelihood. When using 35 rungs, TIDE produces log-marginal likelihoods that are close to those obtained through standard TI and have low variance estimates (SD < 1) after 3000 to 3500 samples. When using 50 rungs, this is achieved in roughly 2000 iterations. In addition, when using a small number of rungs (i.e., 10/20), TIDE more closely matches the marginal likelihood obtained using a higher number of rungs than standard TI. Thus, our results suggest that TIDE provides a promising method of performing TI when estimating marginal likelihoods for models of individual subjects.

### Hierarchical example

As with the individual subjects simulations, we defined both a “simple” and “complex” model. In addition, we defined two other models commonly of interest in applications of evidence accumulation models: a “drift-rate only” model, and a “threshold only” model. Lastly, in addition to defining a “simple” dataset, we also defined a “drift-rate” dataset, which had the same parameters varying across conditions as in the data-generating process of the “drift-rate only” model. The specific model definition for data generation and fitting can be found in the Appendix. For each model fit to each dataset, we used standard TI without random pairing (i.e., dependent sampling between individual- and group-level parameters), standard TI with random pairing (i.e., independent sampling between individual- and group-level parameters), TIDE (i.e., dependent sampling between individual- and group-level parameters), and TIDEz (i.e., independent sampling between individual- and group-level parameters).

**Fig. 3:**
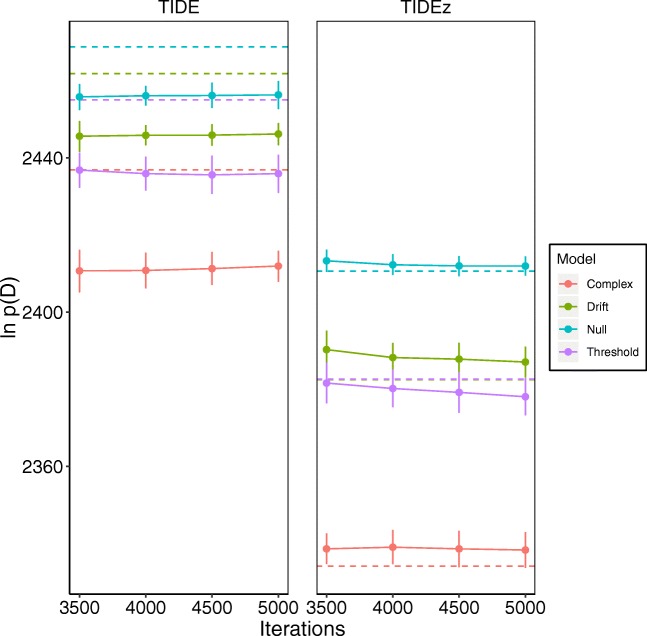
The estimated natural logarithm of the marginal likelihoods (*y*-axis) for each of the different models (*different lines*) across different numbers of samples (*x*-axis; note that this includes the samples discarded for burn-in) on the “simple” dataset. The *left panel* displays TIDE, which uses dependent sampling between the individual-level and group-level parameters, whereas the *right panel* displays TIDEz, which uses independent sampling between the individual-level and group-level parameters. The *dashed lines* display the values obtained through standard TI (fixed number of samples), which differ between panels based on sampling dependency, the *solid lines* with circular points display the values obtained through TIDE/TIDEz. Note that in the right panel the TI estimate for the drift rate model is partially blocked out by the threshold model, as these estimates are very close to one another

**Fig. 4:**
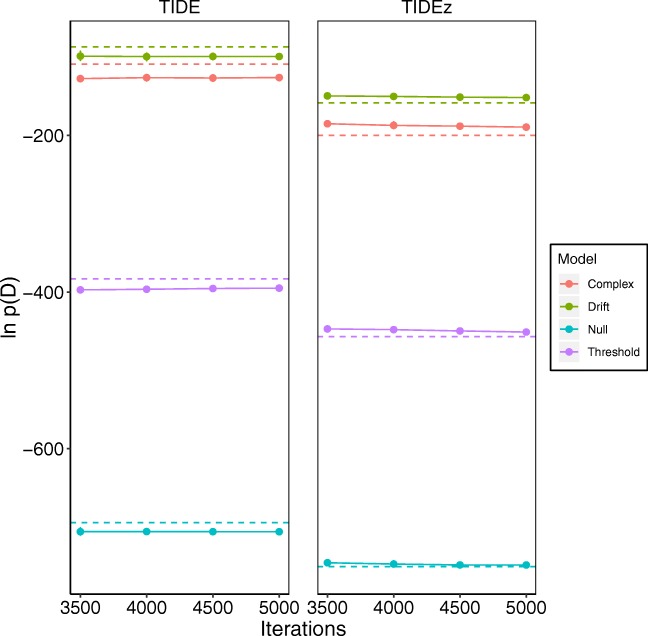
The estimated natural logarithm of the marginal likelihoods (*y*-axis) for each of the different models (*different lines*) across different numbers of samples (*x*-axis; note that this includes the samples discarded for burn-in) on the “drift-rate” dataset. The *left panel* displays TIDE, which uses dependent sampling between the individual-level and group-level parameters, whereas the *right panel* displays TIDEz, which uses independent sampling between the individual-level and group-level parameters. The *dashed lines* display the values obtained through standard TI (fixed number of samples), which differ between panels based on sampling dependency, the *solid lines with circular points* display the values obtained through TIDE/TIDEz

The results of applying these methods can be seen in Fig. 3 for the simple dataset, and Fig. 4 for the drift-rate dataset. In each figure, the left panel displays the methods that use dependent sampling of individual- and group-level parameters, and the right panel displays the methods that use independent sampling. For each method, we used 35 temperature rungs. For TIDE and TIDEz, we discarded the first 3500 samples for each chain (i.e., each temperature) as burn-in, meaning the *x*-axis of Figs. 3 and 4 begin at the end of burn-in. We also ran these methods ten independent times, with points representing means and error bars representing standard deviations. For TI, we used 12 chains for the simple model, 14 chains for the “drift-rate only model, 14 chains for the “threshold only” model, and 18 chains for the complex model (i.e., twice the number of free parameters), with 1500 samples per chain and the first 800 samples per chain discarded as burn-in. In terms of computational workload, TI used 18,000 samples per temperature for the simple model, 21,000 samples per temperature for the drift-rate and threshold only models, and 27,000 samples per temperature for the complex model, whereas TIDE and TIDEz (at the maximum point of the *x*-axis) used 5000 samples per temperature for each model,^3^ meaning that TIDE and TIDEz took 3.6 times (simple model), 4.2 times (drift-rate and threshold only models), and 5.4 times (complex model) less samples than TI in this example (see the Appendix [Equation C3] for a more detailed theoretical comparison of computational workloads).

First, and perhaps most interestingly, the use of dependent or independent sampling has a large effect on the approximated log-marginal likelihood, with dependent sampling resulting in much larger log-marginal likelihoods. This suggests that the seemingly minor change to the dependency of the sampling can have a potentially large impact on the approximated log-marginal likelihood, meaning that these sampling assumption should be carefully considered, and not chosen arbitrarily. However, we believe that the choice between these sampling assumptions is complex, and there is currently no clear recommendation that can be given that covers all potential situations. Specifically, the independent sampling assumption is equivalent to placing a zero probability prior on the correlations between the group and subject level being greater than zero, which in many situations is probably false (i.e., subject-level parameters do correlate with group-level parameters). However, cognitive models often use simplifying assumptions that are potentially false if they endow the model with certain practical advantages, and as discussed previously, the independent-sampling assumption can have several practical advantages for sampling. As a practical recommendation, we suggest that researchers attempt to use the model where correlations between group- and subject-level parameters are explicitly modeled (i.e., dependent sampling). Alternatively, if sampling is poor, then it is reasonable to switch to a model where these correlations are no longer considered (i.e., independent sampling). However, we believe that the potential impact of these different sampling assumptions should be explored in more detail in future research in order to find a more conclusive recommendation.

Second, although all methods (when matched on the dependency assumption) appear to fairly closely agree in most situations, there appears to be some large differences in the approximated log-marginal likelihood for standard TI and TIDE for each of the models in the simple data (Fig. 3, left panel). Interestingly, the model orderings remain the same, but the TIDE marginal likelihoods appear to be some constant factor lower than those obtained from standard TI (i.e., about 30 on the log scale). However, this may not necessarily indicate an inaccuracy of TIDE, as potential sampling problems were the original reason for the switch from dependent sampling to independent sampling, meaning that either (or both) method(s) may be inaccurate with the dependency included. Overall though, TIDE and TIDEz appear to show good agreement with TI in many situations, despite TIDE/TIDEz having fewer samples per temperature than TI, suggesting that TIDE/TIDEz may be promising methods for approximating marginal likelihoods with a reduced computational workload. However, we believe that the discrepancies observed here motivate future work with a large-scale comparison between TI, TIDE, and some of the other marginal likelihood approximation methods (e.g., bridge sampling; Gronau et al., 2017), using both dependence and independence assumptions about the individual- and group-level parameters in order to assess the agreement between methods that are intended to approximate the same quantity.

### Application to empirical data

Although TIDE and TIDEz appear to produce sensible results in simulated environments where the generating process is known, the additional noise and uncertainty of empirical data can result in greater difficulties in selecting between competing models (Evans and Brown, 2018). To see whether empirical data would prove problematic for TIDE/TIDEz, we applied the method to the data of Rae et al., (2014), which have been used in the previous papers of Evans and Brown (2018) and Annis et al., (2018) as a benchmark. For brevity, we will only provide the essential details of the Rae et al., (2014) study here, though interested readers can see more in Rae et al., (2014), Evans and Brown (2018), or Annis et al., (2018).

In the study of Rae et al., (2014), each participant completed a perceptual decision-making task under two different sets of emphasis instructions: speed and accuracy. The key finding of the study was that both drift rate and threshold changed as a function of emphasis, as opposed to previous assumptions that emphasis only influenced threshold. Following Annis et al., (2018), we fit four models to these data: one that allowed no parameters to vary over emphasis, one that allowed only drift rate to vary, one that allowed only threshold to vary, and one that allowed both drift rate and threshold to vary. The exact model definitions are identical to those of Annis et al., (2018). We fit each model using 35 parallel chains (i.e., 35 temperature rungs), with 5000 samples per chain discarded as burn-in, and 3000 samples per chain used to calculated the mean log-likelihood for each temperature.

**Fig. 5:**
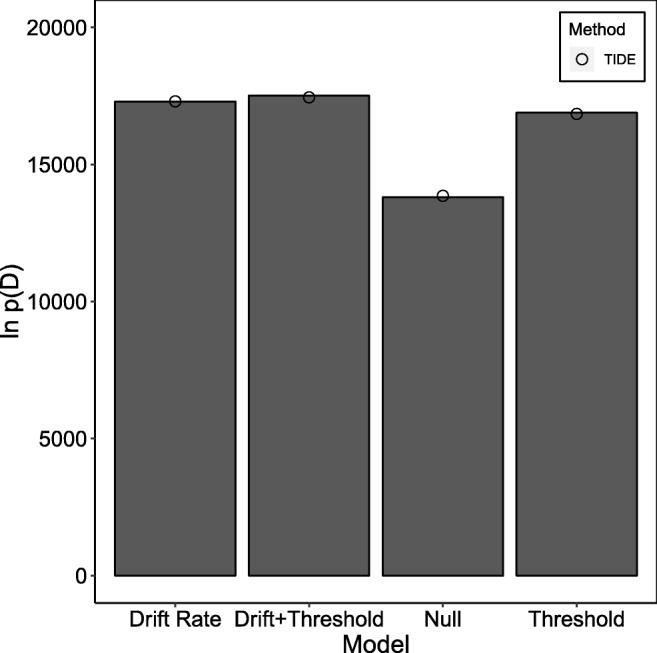
The estimated natural logarithm of the marginal likelihoods (*y*-axis) for each of the different models (*x*-axis) on the dataset of Rae et al., (2014). *Column bars* display the standard TI approximation with dependent sampling between individual- and group-level parameters, and *circles* display the TIDE approximation. *Error bars* for the TIDE approximation have not been included, as they were smaller than the circles used to represent the approximation

**Fig. 6:**
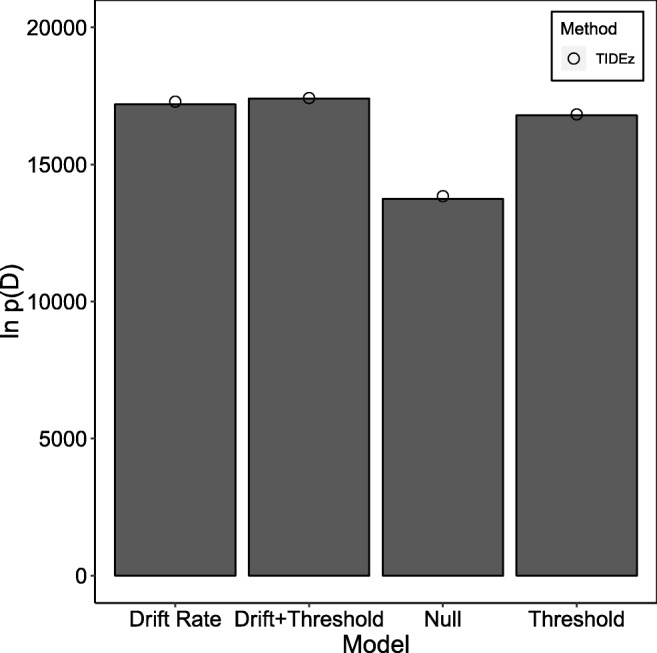
The estimated natural logarithm of the marginal likelihoods (*y*-axis) for each of the different models (*x*-axis) on the dataset of Rae et al., (2014). *Column bars* display the standard TI approximation with independent sampling between individual- and group-level parameters, and *circles* display the TIDEz approximation. *Error bars* for the TIDEz approximation have not been included, as they were smaller than the circles used to represent the approximation

The results of the fits can be seen in Fig. 5 (TIDE) and Fig. 6 (TIDEz). The *x*-axis displays different models, and the *y*-axis displays the estimated log-marginal likelihood, with larger numbers suggesting a better model. We ran ten independent fitting routines for each model, with the column bars (standard TI) and circle (TIDE/TIDEz) on the graph represented the mean estimated marginal likelihood over these ten fits, and error bars being omitted as the standard deviation in the estimate was smaller than the circle marker used to display the means. These results seem to indicate that both TIDE and TIDEz perform well when applied to empirical data: both methods shows little variability in the estimated log-marginal likelihood, and both select the drift rate and threshold model as the best model, with all models in the same ordering as standard TI.

## Discussion

The aim of this article was to provide a simple, computationally efficient method of calculating Bayes factors for complex psychological models with correlated parameters. We proposed TIDE, an extension to TI that integrates the DE-MCMC method through the logic of Calderhead and Girolami’s (2009) TI through population MCMC. As discussed earlier, TI requires many MCMC runs over a set of power posteriors to obtain the marginal likelihood, whereas TIDE only requires a single MCMC run, which can reduce the computational workload. We found that TIDE also closely matches the TI approximation of the log-marginal likelihood for when applied to the data of individual subjects using the LBA (Brown and Heathcote, 2008). However, when applied to hierarchical models, the methods match somewhat less closely, and in one situation standard TI and TIDE show large differences, though by what appears to be some constant offset. We believe that TIDE provides a promising and simple-to-implement method of estimating the marginal likelihood of complex cognitive models, which will likely allow these approximations to be performed with minimal computational resources, such as personal computers. Our code for implementing TIDE in *R* (R Core Team, 2017) can be found at https://osf.io/ntmgw/, though note that the implementation of TIDE from a standard DE algorithm is extremely easy, and only requires assigning a temperature to each chain. We also include all simulated data sets used within this manuscript with the code, as a benchmark for those who wish to check their custom-written TIDE algorithms.

It is also important to note that DE-MCMC contains some unique limitations based on the crossover step used to generated proposals (ter Braak & Vrugt, 2008), which may potentially be solved by TIDE. Specifically, as more chains arrive at the high-density areas of the posterior distribution over the course of sampling, the proposed jump steps more commonly become smaller. When the likelihood function of the model has many peaks and troughs, as is often the case in models with correlated parameters, it can be difficult, or impossible, for a proposal to be made that would result in the remaining chain(s) moving into the posterior region. This can result in a few chains getting “stuck” in regions outside the posterior, meaning that the sampled posterior distribution would be contaminated with some samples that are not truly from the posterior. These problems can be easily spotted through visual inspection of the chains, or standard convergence statistics (e.g., $\hat {R}$; see Gelman & Rubin, (1992)), and can often be overcome with techniques such as “migration” (attempting to exchange parameter values between chains; see Turner et al., (2013)) or variable jump steps (ter Braak and Vrugt, 2008). However, these solutions are not always effective (ter Braak & Vrugt, 2008), and incorrect use of migration can result in convergence to a local maxima, or the sampling of an overly narrow posterior (i.e., not the true posterior distribution). Interestingly, TIDE also provides a potential solution to the “stuck” chain problem of DE-MCMC, as the interaction between temperatures in TIDE produce a natural variability in the size of jumps proposed with each chain estimating a different distribution. This eliminates the need for techniques like “migration” or variable jump steps, meaning that every proposal is based on the same algorithm, and therefore, implementation is more straight-forward. Indeed, we did not use migration for TIDE or TIDEz in any example within this article, and did not appear to encounter any problems with sampling from the correct posterior distributions. Therefore, TIDE may provide some attractive properties beyond a reduction in computational workload.

One broader issue that we have not discussed is whether or not Bayes factors *should* be the method for selecting between cognitive models. Many researchers have suggested that the Bayes factor provides the optimal balance between goodness-of-fit and flexibility in model selection (Kass & Raftery, 1995; Myung & Pitt, 1997; Evans et al., 2017a), and substantial recent research has gone into developing computationally feasible methods for calculating Bayes factors (Wagenmakers et al., 2010; Gronau et al., 2017; Pajor, 2017; Evans & Brown, 2018; Wang et al., 2018). However, the Bayes factor has also been criticized for the computational burden associated with calculating the marginal likelihood, and more importantly for their sensitivity to the specification of the prior distribution (see Vanpaemel, 2010, Lee & Vanpaemel, 2018 for discussions). The Bayes factor is also only one of many possible methods for model selection, with alternatives existing such as the deviance information criterion (DIC; (Spiegelhalter et al., 2002)), the widely applicable information criterion (WAIC; Vehtari et al., 2017, though see Gronau and Wagenmakers (2018) for limitations of leave-one-out cross validation, which WAIC approximates), normalized maximum likelihood (Myung et al., 2006), and proper scoring rules (Dawid & Musio, 2015), just to name a few. However, the aim of our article was only to propose a new method of estimating the marginal likelihood, and not to debate which method(s) of model selection are superior to others. Therefore, we leave debates on which methods should be used over others to future research.

Lastly, it should be made clear that there were several discrepancies, both minor and major, when marginal likelihoods were approximated for hierarchical models. Standard TI and TIDE did not show extremely close agreement in the hierarchical cases—in contrast to the assessment of individual subjects—and the use of dependent vs. independent sampling of individual- and group-level parameters resulted in large differences in the approximated marginal likelihoods. Therefore, we believe that future research should aim to perform a detailed comparison between different methods of estimating marginal likelihoods for cognitive models (including other methods, such as bridge sampling; Gronau et al., 2017), as well as a more detailed assessment of whether sampling assum- ptions can make meaningful differences on inferences bet- ween models, and which assumptions seem most sensible.

## Appendix A: Thermodynamic integration derivation

Thermodynamic integration (TI; Friel & Pettitt, 2008, Lartillot & Philippe, 2006) represents the marginal likelihood as a one-dimensional integral, which can then be estimated using standard numerical integration techniques. In this section, we show the derivation for TI. To begin, the likelihood in the posterior is raised to a power, *t*. This leads to the *power posterior*:

A1 $$ p(\boldsymbol{\theta}|\boldsymbol{D},t) = \frac{p(\boldsymbol{D}|\boldsymbol{\theta})^{t} p(\boldsymbol{\theta})}{p(\boldsymbol{D}|t)} $$

The power posterior has the following marginal likelihood, which we refer to as the *power marginal likelihood*:

A2 $$ p(\boldsymbol{D}|t) = p(\boldsymbol{\theta}|\boldsymbol{D})^{t} p(\boldsymbol{\theta})\ d\boldsymbol{\theta}\ ,t \in [0,1]. $$

Given this formulation of the marginal likelihood, it is possible to write the log of the marginal likelihood as a difference between log-marginal likelihoods at *t* = 1 and *t* = 0, omitting the notation for the model:

A3 $$ \ln p(D) = \ln p(D|t = 1) - \ln p(D|t = 0), $$

where *p*(*D*|*t* = 0) is a prior distribution that integrates to unity (i.e., is proper) and therefore returns zero when the log is taken. We then introduce the following identity:

A4 $$ \ln p(\boldsymbol{D}|t = 1) - \ln p(\boldsymbol{D}|t = 0) = {\int_{0}^{1}} \frac{d}{dt}\ \ln p(\boldsymbol{D}|t)\ dt. $$

Taking the derivative with respect to *t* we find the following:

A5 $$\begin{array}{@{}rcl@{}} \frac{d}{dt}\ln p(\boldsymbol{D}|t) &=& \frac{1}{p(\boldsymbol{D}|t)}\ \frac{d}{dt}\ p(\boldsymbol{D}|t)\\ &=& \frac{1}{p(\boldsymbol{D}|t)}\ \int p(\boldsymbol{D}|\boldsymbol{\theta})^{t}\ \ln p(\boldsymbol{D}|\boldsymbol{\theta})p(\boldsymbol{\theta})\ d\boldsymbol{\theta}\\ &=& \int \frac{p(\boldsymbol{D}|\boldsymbol{\theta})^{t} p(\boldsymbol{\theta})}{p(\boldsymbol{D}|t)}\ \ln p(\boldsymbol{D}|\boldsymbol{\theta})\ d\boldsymbol{\theta}\\ &=& E_{\boldsymbol{\theta}|\boldsymbol{D},t}\ \ln p(\boldsymbol{D}|\boldsymbol{\theta}) \end{array} $$

Substituting this result into Equation A4 we see that the log-marginal likelihood is the integral of the expected posterior deviance from 0 to 1 with respect to the temperature, *t*:

A6 $$ \ln p(\boldsymbol{D}) = {\int_{0}^{1}} \mathbf{E}_{\boldsymbol{\theta}|\boldsymbol{D},t}\ \ln p(\boldsymbol{D}|\boldsymbol{\theta})\ dt $$

This result indicates that the log-marginal likelihood can be expressed as a one-dimensional integral, which can be approximated with standard numerical integration techniques.

### Hierarchical setting

The derivation for TI in the hierarchical setting is straightforward. The hierarchical structure assumed is given by:

A7 $$\begin{array}{@{}rcl@{}} \boldsymbol{D}_{s} &\sim& p(\boldsymbol{D}_{s} | \boldsymbol{\theta}_{s}) \\ \boldsymbol{\theta}_{s} &\sim& p(\boldsymbol{\theta}_{s} | \boldsymbol{\phi}) \\ \boldsymbol{\phi} &\sim& p(\boldsymbol{\phi}), \end{array} $$

where *s* indexes the participant, ***ϕ*** are the group-level parameters, and ***𝜃*** are the individual-level parameters. The power posterior is given by an application of Bayes’ rule:

A8 $$ p(\boldsymbol{\theta},\boldsymbol{\phi}|\boldsymbol{D},t) = \frac{p(\boldsymbol{D}|\boldsymbol{\theta},\boldsymbol{\phi})^{t}p(\boldsymbol{\theta} , \boldsymbol{\phi})}{p(\boldsymbol{D}|t)}. $$

The structure of the hierarchical model is such that the data are conditionally independent of the group-level parameters. This allows us to write the power posterior as:

A9 $$ p(\boldsymbol{\theta},\boldsymbol{\phi}|\boldsymbol{D},t) = \frac{p(\boldsymbol{D}|\boldsymbol{\theta},\boldsymbol{\phi})^{t}p(\boldsymbol{\theta} | \boldsymbol{\phi})p(\boldsymbol{\phi})} {p(\boldsymbol{D}|t)}, $$

where the power marginal likelihood is given by:

A10 $$ p(\boldsymbol{D}|t) = \int\int p(\boldsymbol{D}|\boldsymbol{\theta},\boldsymbol{\phi})^{t}p(\boldsymbol{\theta} | \boldsymbol{\phi})p(\boldsymbol{\phi}) d\boldsymbol{\theta} d\boldsymbol{\phi}. $$

Recall that TI relies on taking the log of the power marginal likelihood and finding its derivative:

A11 $$ \frac{d}{dt} \ln p(\boldsymbol{D}|t) = \int \int \frac {p(\boldsymbol{D} | \boldsymbol{\theta})^{t} p(\boldsymbol{\theta} | \boldsymbol{\phi}) p(\boldsymbol{\phi})} {p(\boldsymbol{D}|t)} \ln p(\boldsymbol{D} | \boldsymbol{\theta}) d \boldsymbol{\theta} d {\boldsymbol{\phi}}. $$

This leads to the following expectation:

A12 $$ \frac{d}{dt} \ln p(\boldsymbol{D}|t) = \mathbf{E}_{\boldsymbol{\theta},\boldsymbol{\phi} | \boldsymbol{D},t} \ln p(\boldsymbol{D} | \boldsymbol{\theta}). $$

Substituting this result into Equation A4 we have the following one-dimensional integral representation of the marginal likelihood:

A13 $$ \ln p(\boldsymbol{D}) = {\int_{0}^{1}} \mathbf{E}_{\boldsymbol{\theta},\boldsymbol{\phi}|\boldsymbol{D},t}\ \ln p(\boldsymbol{D}|\boldsymbol{\theta})\ dt. $$

This implies the computation of the TI marginal likelihood estimate involves sampling from the joint power posterior, *p*(***𝜃***,***ϕ***|***D***,*t*), and then using the individual-level samples, ***𝜃***_*i*_, to compute the average log-likelihood.

## Appendix B: Model definitions

### Individual subjects analysis

The data for this “simple” dataset were generated with the following parameter values:

$$\begin{array}{@{}rcl@{}} v.c &=& 3.5 \\ v.e &=& 1 \\ s.c &=& 1 \\ s.e &=& 1 \\ b - A &=& 0.4 \\ A &=& 1 \\ t0 &=& 0.3 \end{array} $$

where .*c* and .*e* refer to the accumulators for correct and error responses, respectively.

The “simple” model had the following prior distributions:

$$\begin{array}{@{}rcl@{}} v.c &\sim& N_{+}(3,3) \\ v.e &\sim& N_{+}(1,1) \\ s.e &\sim& N_{+}(1,1) \\ b - A &\sim& N_{+}(0.4,0.4) \\ A &\sim& N_{+}(1,1) \\ t0 &\sim& N_{+}(0.3,0.3) \end{array} $$

where *s*.*c* was fixed to 1 to satisfy a scaling property in the model (Donkin et al., 2009), and *N*_+_ refers to the normal distribution truncated to only positive values. The “complex” model had the following prior distributions:

$$\begin{array}{@{}rcl@{}} v.c_{j} &\sim& N_{+}(3,3) \\ v.e &\sim& N_{+}(1,1) \\ s.e &\sim& N_{+}(1,1) \\ b_{j} - A &\sim& N_{+}(0.4,0.4) \\ A &\sim& N_{+}(1,1) \\ t0_{j} &\sim& N_{+}(0.3,0.3) \end{array} $$

where *j* indexes the condition.

### Hierarchical analysis

The data for the hierarchical analysis were generated using distributions of parameter values, where each of the 10 simulated subjects had parameter values randomly drawn from these distributions. For the “simple” dataset, the following distributions were used:

$$\begin{array}{@{}rcl@{}} v.c_{i} &\sim& N_{+}(3.5,0.35) \\ v.e_{i} &\sim& N_{+}(1,0.1) \\ s.e_{i} &\sim& N_{+}(1,01) \\ b_{i} - A_{i} &\sim& N_{+}(0.4,0.04) \\ A_{i} &\sim& N_{+}(1,01) \\ t0_{i} &\sim& N_{+}(0.3,0.03) \end{array} $$

where *i* indexes the participant, and for the “drift-only” dataset, the following distributions were used:

A14 $$\begin{array}{@{}rcl@{}} v.c_{i,cond1} &\sim& N_{+}(4,0.4) \\ v.c_{i,cond2} &\sim& N_{+}(3,0.3) \\ v.e_{i} &\sim& N_{+}(1,0.1) \\ s.e_{i} &\sim& N_{+}(1,0.1) \\ b_{i} - A_{i} &\sim& N_{+}(0.4,0.04) \\ A_{i} &\sim& N_{+}(1,0.1) \\ t0_{i} &\sim& N_{+}(0.3,0.03) \end{array} $$

where *c**o**n**d*1 and *c**o**n**d*2 are the two “within-subjects” conditions.

All models had the following prior distributions in common:

$$\begin{array}{@{}rcl@{}} v.e_{i} &\sim& N_{+}(\mu_{v.e},\sigma_{v.e}) \\ s.e_{i} &\sim& N_{+}(\mu_{s.e},\sigma_{s.e}) \\ A_{i} &\sim& N_{+}(\mu_{A},\sigma_{A}) \\ \mu_{v.e},\mu_{s.e},\mu_{A} &\sim& N_{+}(1,1) \\ \sigma_{v.e},\sigma_{s.e},\sigma_{A} &\sim& N_{+}(1,1) \end{array} $$

the “simple” model had the following prior distributions:

$$\begin{array}{@{}rcl@{}} v.c_{i} &\sim& N_{+}(\mu_{v.c},\sigma_{v.c}) \\ t0_{i} &\sim& N_{+}(\mu_{t0},\sigma_{t0}) \\ b_{i} - A_{i} &\sim& N_{+}(\mu_{b},\sigma_{b}) \\ \mu_{v.c},\sigma_{v.c} &\sim& N_{+}(3,3) \\ \mu_{t0},\sigma_{t0} &\sim& N_{+}(0.3,0.3) \\ \mu_{b},\sigma_{b} &\sim& N_{+}(0.4,0.4) \end{array} $$

the “drift-rate only” model had the following prior distributions:

$$\begin{array}{@{}rcl@{}} v.c_{i,j} &\sim& N_{+}(\mu_{v.c_{j}},\sigma_{v.c_{j}}) \\ t0_{i} &\sim& N_{+}(\mu_{t0},\sigma_{t0}) \\ b_{i} - A_{i} &\sim& N_{+}(\mu_{b},\sigma_{b}) \\ \mu_{v.c_{j}},\sigma_{v.c_{j}} &\sim& N_{+}(3,3) \\ \mu_{t0},\sigma_{t0} &\sim& N_{+}(0.3,0.3) \\ \mu_{b},\sigma_{b} &\sim& N_{+}(0.4,0.4) \end{array} $$

where *j* indexes the condition, the “threshold only” model had the following prior distributions:

$$\begin{array}{@{}rcl@{}} v.c_{i} &\sim& N_{+}(\mu_{v.c},\sigma_{v.c}) \\ t0_{i} &\sim& N_{+}(\mu_{t0},\sigma_{t0}) \\ b_{i,j} - A_{i} &\sim& N_{+}(\mu_{b_{j}},\sigma_{b_{j}}) \\ \mu_{v.c},\sigma_{v.c} &\sim& N_{+}(3,3) \\ \mu_{t0},\sigma_{t0} &\sim& N_{+}(0.3,0.3) \\ \mu_{b_{j}},\sigma_{b_{j}} &\sim& N_{+}(0.4,0.4) \end{array} $$

and the “complex” model had the following prior distributions:

$$\begin{array}{@{}rcl@{}} v.c_{i,j} &\sim& N_{+}(\mu_{v.c_{j}},\sigma_{v.c_{j}}) \\ t0_{i,j} &\sim& N_{+}(\mu_{t0_{j}},\sigma_{t0_{j}}) \\ b_{i,j} - A_{i} &\sim& N_{+}(\mu_{b_{j}},\sigma_{b_{j}}) \\ \mu_{v.c_{j}},\sigma_{v.c_{j}} &\sim& N_{+}(3,3) \\ \mu_{t0_{j}},\sigma_{t0_{j}} &\sim& N_{+}(0.3,0.3) \\ \mu_{b},\sigma_{b} &\sim& N_{+}(0.4,0.4) \end{array} $$

## Appendix C: Theoretical comparison of computational workloads of TIDE and TI

In this section, we compare, in a theoretical manner, the computational workloads of TIDE and standard TI. The key advantage of TIDE over standard TI is that it only requires a single chain per power posterior.

When assuming that TI and TIDE are implemented with identical MCMC samplers (i.e., DE-MCMC) where each sample takes an identical time (on average) to evaluate, and only a single computational core is available (i.e., no ability for multi-core parallelization), computational workload depends on only the number of samples that need to be taken for each method, and can be calculated by:

C1 $$ CW = \frac{[B_{ti} + S_{ti}] \times C_{ti} \times T_{ti}}{[B_{tide} + S_{tide}] \times C_{tide} \times T_{tide}} $$

where *C**W* is the computational workload of TI relative to TIDE, *B* is the number of burn-in samples per chain, *C* is the number of chains per temperature, *T* is the number of temperatures used, and *S* is the number of samples required from the posterior per chain to create the integration curve. Specifically, *C**W* is how many times less computationally taxing TIDE is than TI, meaning that values of *C**W* greater than 1 indicate that TIDE is less computationally taxing than TI. In the case of TIDE, the method only requires one chain per temperature (i.e., *C*_*t**i**d**e*_ = 1), meaning that Eq. C1 can be simplified to:

C2 $$ CW = \frac{[B_{ti} + S_{ti}] \times C_{ti} \times T_{ti}}{[B_{tide} + S_{tide}] \times T_{tide}} $$

When assuming that TI and TIDE are implemented with an equal number of temperatures, Eq. C2 can be simplified again:

C3 $$ CW = \frac{[B_{ti} + S_{ti}] \times C_{ti}}{[B_{tide} + S_{tide}]} $$

which essentially means that whenever the total number of samples in the TI algorithm (i.e., *B*_*t**i*_ + *S*_*t**i*_) is greater than the ratio of the total number of samples in the TIDE algorithm to the number of chains in the TI algorithm (i.e., $\frac {B_{tide} + S_{tide}}{C_{ti}}$), then TIDE will be less computationally taxing than TI. Importantly, the number of chains for the standard DE-MCMC algorithm (used in TI for this situation) is commonly set to 3*k*, where *k* is the number of free parameters, meaning that TIDE will become decreasingly computationally taxing compared to TI when the dimensionality of the model increases.

Introducing the potential for multiple processing cores, and therefore, cross-core parallelization, makes the computational workload calculation somewhat more complicated. In cases where the number of computational cores is less than or equal to the number of temperatures used, and the number of temperatures can be equally divided into computational cores (i.e., no remainder from the equation $\frac {T_{ti}}{nCPU}$, where *n**C**P**U* is the number of computing cores), TI provides the ability for completely independent parallelization, where parallelization can be used without any cost in computational workload. Although TIDE can be parallelized across chains within each iteration of the sampling algorithm, this type of parallelization still has some level of dependency (i.e., only one iteration can be done in parallel at a time), meaning that there is some cost in computational workload when using multiple cores for TIDE, which will differ from situation to situation. This can be reflected by re-writing Eq. C3 as:

C4 $$ CW = \frac{([B_{ti} + S_{ti}] \times C_{ti})/nCPU}{([B_{tide} + S_{tide}])/(nCPU \times P_{tide})} $$

where *P* is a coefficient for how imperfect the TIDE parallelization is, with 0 reflecting no benefit of parallelization, and 1 reflecting perfect parallelization. Importantly, when *P*_*t**i**d**e*_ is small and *n**C**P**U* is large, TIDE can be more computationally taxing than TI, meaning that in situations where multiple cores are available these factors should be considered.

Lastly, using a sampler other than DE-MCMC (e.g., sampling the model using the software Stan (Gelman et al., 2015), see Annis et al., (2017) for an example) for the TI estimate also makes the computational workload calculation somewhat more complicated. Importantly, we can no longer assume that samples take the same amount of time to generate, meaning that computational workload cannot be purely expressed in terms of the number of samples required to approximate the integration curve. This can be reflected by re-writing Eq. C3 as:

C5 $$ CW = \frac{[B_{ti} + S_{ti}] \times C_{ti} \times t_{ti}}{[B_{tide} + S_{tide}] \times t_{tide}} $$

where *t* is the average time taken to generate a sample in each of the sampling algorithms. Importantly, this means that the computational workload of TI can be reduced by using a sampler that either (1) decreases the number of chains required without proportional increases to the time per sample, or (2) decreases the time per sample without proportion increases to the number of chains required.

## Footnotes

1 As far as we are aware, this solution was first suggested by Brandon M. Turner.

2 Running TIDE for 5000 samples on a single CPU core of a Mac OS laptop with a 1.7 GHz Intel Core i7 processor and 8 GB 1600-MHz DDR3 memory took approximately 4 min for ten temperature rungs, and approximately 19 min for 50 temperature rungs.

3 Running TIDE/TIDEz for 5000 samples on a single CPU core of a Mac OS laptop with a 1.7-GHz Intel Core i7 processor and 8 GB 1600-MHz DDR3 memory took approximately 77 min for 35 temperature rungs.
